# Flash-comet assay

**DOI:** 10.1016/j.mex.2020.101161

**Published:** 2020-11-27

**Authors:** Erik Bivehed, Björn Hellman

**Affiliations:** Uppsala University, Department of Pharmaceutical Biosciences, Drug Safety and Toxicology, Uppsala, Sweden

**Keywords:** DNA-damage, Gel electrophoresis, Genotoxicity testing, Gentle lysis, In vitro comet assay, Low conductivity electrophoresis solution, Single cell analysis

## Abstract

In the present paper, we present a substantially revised protocol of the widely used SCGE assay performed under alkaline conditions. In our updated version of the comet assay, which we call the Flash-comet, LiOH is used instead of NaOH during the unwinding and electrophoresis. This allows a higher voltage during the electrophoresis (5 V/cm instead of 0.7 V/cm), making it possible to reduce the unwinding time from 20 to 40 to 2.5 min, and the electrophoresis time from 10 to 20 to 1 min. Still, the Flash-comet was found to detect DNA strand breaks and alkali-labile sites with a higher degree of sensitivity than the conventional protocol in cells that had been exposed to H_2_O_2_ or ionizing radiation. In order to prevent alkaline hydrolysis of DNA, the wash and lysis solutions have been modified in the Flash-comet protocol.

• By using an alkaline LiOH-based medium, the Flash-comet allows for much shorter times for both unwinding and electrophoresis than the conventional comet assay without compromising the sensitivity.
• The reduced run-times of the unwinding and electrophoresis steps in the Flash-comet should also reduce the risk of laboratory-induced alkaline hydrolysis of DNA when evaluating the potential DNA-damaging effects of different types of xenobiotics.

## Graphical abstract

**Figure:**
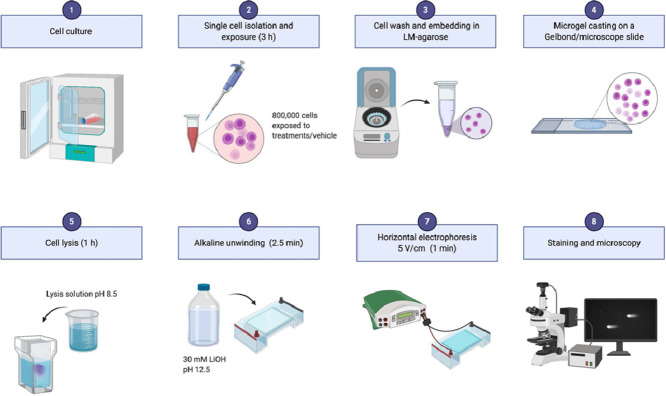

Specifications Table

**Table:** 

Subject Area:	Pharmacology, Toxicology and Pharmaceutical Science
More specific subject area:	*Genetic toxicology*
Method name:	*Flash-comet*
Name and reference of original method:	*Alkaline single cell gel electrophoresis assay (SCGE)/Alkaline comet assay (1,2)*
Resource availability:	

## Method details

The general structure of Flash-comet assay is similar to that of the single cell gel electrophoresis assay, originally presented by Östling and Johanson and performed under neutral conditions [1]. The original protocol was later modified by Singh et al. who performed the assay under alkaline conditions [2]. In comet assay (conventional and Flash), the cells are exposed to xenobiotics when the cells are dispersed in medium. After exposure, the cells are embedded in low-melting point agarose on microscope slides or on GelBond^Ⓡ^ films. The slides/films are then submerged in a cold lysis solution. This solution contains detergents and highly concentrated salt solution in order to disrupt cellular membranes and proteins leaving a protein depleted nucleoid. The samples are then subjected to alkali unwinding in an electrophoresis solution (pH >13) in order to generate relaxation of the super-coiled DNA structure to release DNA-loops and fragments [2], [3], [4], [5]. After unwinding, electrophoresis is performed by applying a strong electric field which will cause migration of the fragmented and relaxed negatively-charged DNA towards the anode [6]. The amount of DNA which migrates towards the anode will depend on the severity of the DNA-damage in the cell. After electrophoresis and staining, a cell with significant DNA-damage (DNA strand breaks) will look like a comet in the microscope. The level of DNA-damage is usually evaluated by measuring the fluorescence intensity of the comet using imaging software (Comet assay IV; Perepective instruments, UK). There are several parameters that could be used, but the most common is probably the percentage of DNA in the tail (% TDNA).

In the present paper, we describe a rather extensively modified protocol of the alkaline version of the comet assay, which we call the Flash-comet assay. The major alteration of the protocol compared to conventional one, is the introduction of a low-conductive electrophoresis solution (30 mM LiOH; pH 12.5), allowing the electrophoresis to be performed at six-times higher voltage. The increased voltage results in an increased sensitivity of the assay and also reduces the electrophoresis run-time from 10 to 20 min to 1 min. Furthermore, due to the high voltage used, the unwinding time can also be reduced from 20 to 40 min down to 2.5 min. The composition of the wash and lysis solutions is also modified in the Flash-comet, mainly by lowering the pH from 10 to 8.5 in order to prevent assay-induced alkaline hydrolysis and DNA denaturation.

## Method procedure

### Equipment and consumables

- Analytical balance
- pH meter
- Centrifuge (preferable with cooling)
- Microwave oven
- Magnetic stirrer
- Horizontal submarine gel electrophoresis tank (large size; gel platform *L* × *W*: 20 × 20 cm; Buffer volume: >2000 ml)
- Adjustable power supply
- Glass beakers
- Erlenmeyer flasks
- Measuring glass
- Staining glass cuvettes
- Slide tray
- Staining box (amber color)
- Glass slides (76 × 26 mm)
- Cover slips (24 × 60 mm)
- Cover slips (50 × 60 mm)
- GelBond^Ⓡ^ film
- Blunt forceps
- Micropipettes
- Eppendorf tubes (1.5 ml and 5 ml)
- Slide storage box
- Fluorescence microscope
- Charge-coupled device (CCD) camera
- Analysis software – Comet Assay IV (Perspective Instruments, UK) or similar

## Stock, ready-to-use and working solutions

The only solutions that are bought as ready-to-use are the cell growth media, supplements and antibiotic for cell culture (requirments vary between cell typea) and phosphate-buffered saline (PBS). All other solutions must be prepared or modified in-house. Some of them can be stored for months, either at room temperature (RT) or at 4 °C. The working solutions must be prepared on the same day the assay is performed.

### Wash solution, 1000 ml, pH 8.5: (1 mM KH_2_PO_4_, 155 mM NaCl Na_2_HPO_4_–7H_2_O, adjusted with NaOH to pH 8.5)

- Mg- and Ca-free PBS
- Adjust pH to 8.5 using NaOH
- Store at 4 °C (can be stored for months under this condition)

#### Lysis solution, pH 8.5

- NaCl (pH 8.5) 5 M (can be stored at RT for months)
- EDTA (pH 8.5) 0.25 M (can be stored at RT for months)
- Tris base (pH 8.5) 250 mM (can be stored at RT for months)
- Triton X-100 or equivalent detergent
- DMSO

#### Lysis working solution, 150 ml: (2.5 mM NaCl, 100 mM Na_2_-EDTA, 10 mM Tris, 1% Triton X-100, 5% DMSO and adjusted with NaOH to pH 8.5)

- 75 ml NaCl, 5 M, pH 8.5
- 60 ml EDTA, 0.25 M, pH 8.5
- 7.5 ml DMSO
- 1.5 ml Triton X-100 (or equivalent detergent)
- 6.0 ml 250 mM Tris adjusted to pH to 8.5 using NaOH
- Cool down to 4 °C

### Electrophoresis solution, pH 12.8

- LiOH (pH 12.8) 3–4 M (can be stored at RT for months)

#### Electrophoresis working solution, 2000 ml, pH 12.5: (30 mM LiOH, pH 12.5)

- 15 ml 4 M LiOH
- Dilute to 2000 ml using deionized H_2_O
- Cool down to 4 °C

### Neutralization buffer, 1000 ml, pH 7.5: (0.4 M Tris-base, adjusted with HCl to pH 7.5)

- Tris-base 0.4 M in H_2_O
- Adjust pH to 7.5 using HCl
- Dilute with H_2_O to 1000 ml
- Store at 4 °C (can be stored for months)

## Slide preparation

This is not an absolute requirement and can be omitted or modified depending on the lab set-up and preference. However, this part of the assay can preferably be conducted in advance in large batches. In order to minimize bias, we recommend to assign a number or letter to each slide and randomly assign them to treatment groups. For example, a full experiment with seven treatment groups requires 21 slides (i.e. three slides per group). The use of GelBond^Ⓡ^ is not necessary although it highly improves gel stability and assures firm adherence. Once prepared the slides are best stored in a dry and dust-free environment.

1. Cut sheets of GelBond^Ⓡ^ (Lonza, Switzerland) into 25 × 55 mm strips.
2. Add a small amount of the glue Norland Optical Adhesive 68 (Thorlabs Inc., US-NJ) to the edge of the frosted part of a microscope slide (26 × 76 mm)
3. Place the Gelbond^Ⓡ^ strip on the microscope slide with the hydrophobic side down (water will bead up on the hydrophobic side)
4. Apply light pressure to ensure that both Gelbond^Ⓡ^ and microscope slide are connected via the glue
5. Irradiate the slide with UV-light (365 nm) for 20 s.
6. Repeat steps 2–5 for all of the slides.

## PBS-agarose for single cell entrapment

The agarose is dissolved in Mg- and Ca Mg- and Ca-free PBS (pH 7.4), in order to maintain ionic pressure and to prevent triggering premature cell death. The stock solution can be aliquoted and stored at 4 °C for several months.

1. In a small Erlenmeyer flask, add low-melting point agarose with Mg- and Ca-free PBS (pH 7.4) to a final concentration of 0.6% (w/w). Make sure to record the total weight.
2. Heat the mixture in a microwave (150 W for 3 min, avoid boiling) until the agarose has dissolved completely.
3. Let the solution cool down to 37 °C. Weigh the flask again and add deionized H_2_O for adjustment to the original weight.

## Pre-assay preparations

1. Pre-heat water bath and heat plate to 37 °C
2. Start warming cell growth medium
3. Prepare lysis and electrophoresis working solutions and cool to 4 °C
4. Melt one aliquot of PBS-agarose (microwave 300 W for approx. 1 min) and place it in the water bath (make sure to close the lid to prevent dilution due to condensation)
5. Pre-cool enough cuvettes enough to host all slides and the horizontal electrophoresis chamber to 4 °C

## Cell exposure

The exposure conditions given below describe a control experiment for suspension cells and may be modified according to required experimental set-up. It should be noted that the exposure conditions may vary depending on the purpose of the study, e.g., the type of cells used (cell line or primary cells, adherent or suspension), with or without metabolic activation, enzymatic treatments and exposure times.

1. Count the cells and determine the cell viability using trypan blue exclusion; >95% is minimum level at this point.
2. Transfer to 1.5 ml test tubes (1 × 10^6^ cells/tube)
3. Bring the total volume to 990 µl using cell growth medium
4. Add 10 µl exposure solution and incubate the tubes at 37 °C with slight agitation for 3 h. If hydrogen peroxide, ionizing radiation or other compound is used as positive exposure, incubation time may be adjusted accordingly.

## Flash-comet

All steps from this point should be performed in an UV-light protecting environment (for example, using a dark room and security light) as fast as possible while keeping the samples refrigerated (not above 4 °C).

1. After exposure, transfer the cells to 4 ml of pre-cooled wash solution.
2. Centrifuge the cells at 230 G (rpc) for 5 min. Remove the supernatant and add 5 ml of cold wash solution. **Note**: Since the exposure medium may contain high levels of agents classified as carcinogenic, mutagenic and/or toxic to reproduction (CMR agents), it should be collected and disposed as hazardous waste.
3. Centrifuge the cells at 230 G (rpc) for 5 min. Add 1 ml of cold wash solution, count the cells and determine the cell viability. **Note**: We usually set the lower limit of viability to 80% using the trypan blue dye-exclusion assay. However, this limit should be regarded as a recommendation rather than a requirement. The acceptable level of cytotoxicity may vary depending on the type of genotoxic exposure (sometimes it is nesseary to allow higher cytotoxicity in order to detect genotoxic agents) and also depending on what type of cytostatic parameter that is used**.** Add 4 ml of cold wash solution and centrifuge the cells at 230 G (rpc) for 5 min.
4. Remove supernatant and add calculated volume of wash solution so that cell concentration is 0.8 × 10^6^ (approx. 100 µl, but never below 50 µl)
5. When all samples are diluted, place them in a water bath at 37 °C for approx. 2–5 min.
6. Aspirate the cell suspension 2–4 times and transfer 30 µl of the cell suspension into a tube containing 210 µl agarose (do not mix, just transfer).
7. Aspirate once and transfer 60 µl of agarose/cell suspension to a pre-heated randomized Gelbond^Ⓡ^ coated microscope slide. Cover slide with a cover slip (24 × 40 mm)
8. Place the slide in the refrigerator or on a cold surface for 1–15 min to allow agarose to set. (If using GelBond^Ⓡ^, this step can be reduced to 1 min)
9. When the agarose has formed a gel, remove the cover slip and place the slide in one of the pre-cooled cuvettes. Add cold lysis solution and incubate for 1 h at 4 °C.
10. After cell lysis, transfer the slide to the horizontal electrophoresis chamber at 4 °C. Make sure to place the gel-side up.
11. Cover the slide with cold electrophoresis solution and incubate for 2.5 min. After incubation, run electrophoresis for 1 min at 150 V (5 V/cm) at 4 °C.
12. After electrophoresis transfer the cells to a horizontal neutralization chamber and cover the slides with cold neutralization solution and incubate for 15 min at 4 °C.
13. After neutralization, dehydrate the gels by incubating in EtOH (70%:95%:99%; 2 min/step) and let dry.
14. Store the slides in a dark, dry and dust-free environment until analysis.

## Staining

In order to prevent fading and to achieve the best staining, it is recommended that the staining is conducted in an UV light protective environment and away from direct light exposure.

1. Rehydrate the slides in 0.4 M Tris-HCL, pH 7.5 for 15 s
2. Add 20 µl of fluorescent stain (e.g. SYBR^Ⓡ^ Gold; 1:10,000 in 0.4 M Tris-HCL) and incubate for 10 min in a humidity chamber
3. Wash slides 2 × 1 min in 0.4 M Tris-HCL
4. Dab off excess liquid with paper and cover gel with cover slips (24 × 55 mm). Store in a humidity chamber to prevent drying.

## Microscopy

In order to prevent fading and to achieve the best staining, it is recommended that the microscopy is conducted in an UV light protective environment and away from direct light exposure.

1. Analyze the slides in chronological order according to the randomization method (A-U; 1–21 or similar)
2. Analysis starts at approx. 3 mm away from the slide edge and follows the midline direction (see Fig. 1) until approx. 3 mm from the gel edge.

**Fig. 1:**
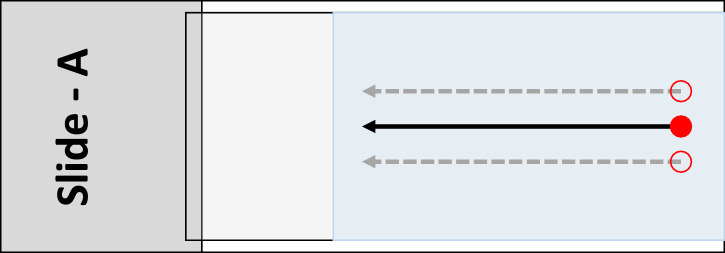
Analysis sweep over a comet slide.
3. Fifty randomly chosen nuclei/comets are scored on each slide. Aside from the parameters that are automatically recorded for each measurement, it is also important to document the number of hedgehogs/clouds/ghosts, the background appearance (clean or cluttered), cell density (high or low), cell aggregation (high or low) and comet tail appearance (brush, granular, smooth, fibrous).
4. If fewer than 50 comets are scored in one sweep, return to starting position and move one frame up/down, represented by the dashed arrows in Fig. 1 (to avoid having the same cells scored twice).

## Method validation

**Fig. 2:**
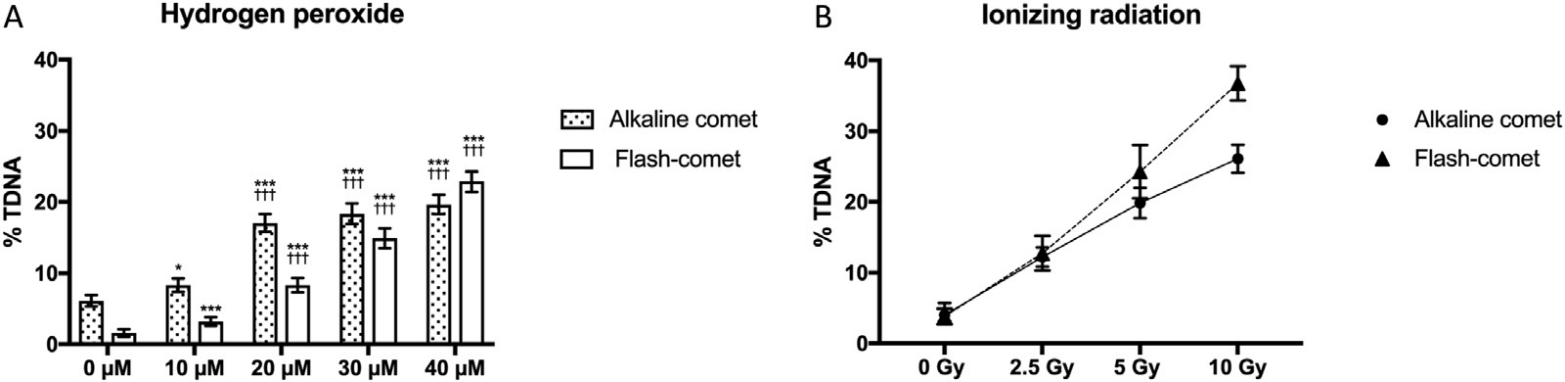
**Panel A:**TK-6 cells were exposed for 15 min to different concentration of either hydrogen peroxide (10–40 µM) or Milli-Q water as the negative control. After exposure, the cells were subjected to either the Flash-comet (white bars) or the conventional alkaline comet assay (dotted bars). The% TDNA was used as the indicator of DNA-damage. The data is presented as means (± 95% confidence interval; CI) after pooling the data from three independent experiments (*n* = 450 cells). Two different methods were used for the statistical analysis (i) a recently published proportional odds model tailored to continuous outcomes which we have called UCDAS (8) (* *P* < 0.05; *** *P* < 0.001) and (ii) a traditional one-way ANOVA with Tukey post-hoc test (††† *P* < 0.001). **Panel B:** In a proof of concept experiment, the% TDNA  was measured using the Flash-comet (triangles) or the conventional alkaline comet assay (circles) on TK-6 cells irradiated with X-rays corresponding to 0, 2.5, 5 and 10 Gy. Both protocols showed a clear dose-response relationship in DNA-damage corresponding to an increased level of irradiation but the slope of dose-response curve for the Flash-comet showed a steeper incline. The data is presented as means (± 95% CI) after pooling the data from three slides (*n* = 150 cells for each exposure).

In a recently accepted paper in Mutation Research [7], our new Flash-comet and the conventional comet assay was used when evaluating the DNA damage induced by hydrogen peroxide and ionizing radiation. As expected, both protocols the conventional comet assay and the Flash-comet assay showed a statistically significant increase in H_2_O_2_-induced DNA damage at all concentrations tested, evaluating the statistical significance using either our recently published proportional odds model tailored to continuous outcomes, which we have called UCDAS [8], or a more traditional, one-way ANOVA. However, whereas the Flash-comet showed a clear concentration dependent increase in the H_2_O_2_-induced DNA-damage, the increase in DNA-damage reached a plateau at 20 μM H_2_O_2_ using the conventional protocol (Fig. 2A). The background level of DNA-damage was also lower in the Flash-comet than in the traditional comet assay. Both versions of the comet assay showed a clear dose response for radiation-induced radiation (Fig. 2B), but the slope of the dose-response curve in the Flash-comet showed a steeper incline compared to what was seen in the conventional assay.

In the present paper, we present a protocol that we have called the Flash-comet; a new alternative approach to the conventional comet assay under alkaline conditions. The most profound modification of the protocol for the Flash-comet is the introduction of a low-conductive electrophoresis solution based on LiOH at pH 12.5. This allows the Flash-comet assay to be conducted at six-times higher voltage compared to the conventional comet assay, leading to a dramatically reduced electrophoresis time and, as it seems, improved sensitivity. However, we do not claim that the times given in the various steps in the Flash-comet protocol are absolutely optimal and we do recognize that the Flash-comet assay also has to be further evaluated using other positive controls than H_2_O_2_ and ionizing radiation.

## Declaration of Competing Interest

The authors declare that they have no known competing financial interests or personal relationships that could have appeared to influence the work reported in this paper.
